# Urinary kidney injury molecule-1 is related to pathologic involvement in IgA nephropathy with normotension, normal renal function and mild proteinuria

**DOI:** 10.1186/1471-2369-15-107

**Published:** 2014-07-07

**Authors:** Peng-cheng Xu, Li Wei, Wen-ya Shang, Shun-li Tian, Dong-mei Gu, Tie-kun Yan, Shan Lin

**Affiliations:** 1 Department of Nephrology, General Hospital of Tianjin Medical University, Tianjin 300052, China; 2 Department of Geratology, General Hospital of Tianjin Medical University, Tianjin 300052, China; 3 Department of Clinical Laboratory, General Hospital of Tianjin Medical University, Tianjin 300052, China

**Keywords:** KIM-1, IgA nephropathy, Oxford classification

## Abstract

**Background:**

IgA nephropathy (IgAN) may progress to renal failure for some patients without any clinical risk factors and it is not unusual to find severe pathologic damage in clinically mild IgAN. We therefore investigated whether urinary kidney injury molecule-1 (KIM-1) was related to pathologic involvement in clinically mild IgAN.

**Methods:**

Urinary KIM-1/creatinine of 51 IgAN patients with normotension, normal renal function and proteinuria < 1.0 g/24 h were tested. Relationships between urinary KIM-1 and pathologic features were analyzed.

**Results:**

Eighteen of the 51 patients had elevated urinary KIM-1. The tubular atrophy/interstitial fibrosis was more severe in patients with elevated urinary KIM-1 than that in patients with normal urinary KIM-1 (T0/T1/T2, 13/5/0 vs. 33/0/0, P = 0.004). Proportion of glomeruli containing cresecents was higher in patients with elevated urinary KIM-1 than that in patients with normal urinary KIM-1 (50% vs. 18%, P = 0.026). Urinary KIM-1 correlated with the proportion of total crescents (R = 0.303, p = 0.031) and fibrous crescents (R = 0.456, p = 0.001), but did not correlate with the proportion of cellular crescents or fibrocellular crescents. Although the proportion of vascular lesions was higher in patients with elevated urinary KIM-1 (44.4%) than that in patients with normal urinary KIM-1 (18.1%), the difference was not significant (p = 0.057). There was no difference of the response to treatment between patients with and without elevated urinary KIM-1 during a short-term follow-up.

**Conclusions:**

Urinary KIM-1 is a reflection of tubularinstitial injury. For patients with clinically mild IgAN, high urinary KIM-1 is related to relatively severe pathologic involvement on renal biopsy.

## Background

Immunoglobulin A nephropathy (IgAN) which is the most common form of primary glomerulonephritis, is clinically heterogeneous and 20–40% of patients will progress to end-stage renal disease (ESRD) eventually after diagnosis [1,2]. Some clinical risk factors have been reported to link with progressive IgAN including hypertension, proteinuria >1.0 g/day, and reduced glomerular filtration rate (GFR), but it is not unusual to find patients with significant histological damage despite the absence of clinical risk factors [3-7]. Since IgAN may be progressive for some patients without clinical risk factors [8], renal biopsy seems to be needed for all patients with suspected IgAN [9]. However, renal biopsy is an invasive examination and not all the patients with clinically trivial disease can be persuaded to accept renal biopsy, so a noninvasive biomarker for assessment of the severity of pathologic involvement is needed.

Tubular/interstitial injury has been indicated to have predictive value of the prognosis of IgAN [10-13]. Kidney injury molecule-1 (KIM-1) is a protein expressed on renal proximal tubule epithelial cells and is a sensitive biomarker for proximal tubule injury [14,15]. Urinary KIM-1 has been reported to be associated with disease severity and predict treatment response in IgAN [16-19]. However, previous studies mainly focused on the value of the urinary KIM-1 in the prediction of renal survival and all these studies involved patients with IgAN from clinically trivial to very severe. Thus, the value of urinary KIM-1 in IgAN without clinical risk factors is unclear. We hypothesize that urinary KIM-1 might be a valuable biomarker indicating significant histological damage in clinically trivial patients. In the current study, we detected the levels of urinary KIM-1 in 51 IgAN patients with normotension, normal renal function and mild proteinuria, and analyzed the relationships between urinary KIM-1 and pathologic variables identified by the recently published Oxford classification of IgAN.

## Methods

### Selection of patients

Fifty-one patients, aged >14 years, with renal-biopsy-proven IgAN, from Tianjin Medical University General Hospital between October 2011 and November 2013 were enrolled. Normotension was defined as systolic blood pressure <140 mmHg and diastolic blood pressure <90 mmHg; Normal renal function was defined as serum creatinine < upper limit of test (100 μmol/L); Mild proteinuria was defined as proteinuria <1 g/24 h. Clinical and laboratory data were collected at the time of renal biopsy. Patients with secondary causes of mesangial IgA deposits, such as Henoch–Schonlein purpura or with comorbid conditions such as diabetes mellitus, liver disease or systemic lupus erythematosis, were excluded from this study. Twenty healthy volunteers were used as normal control. To analyze the relationship between urinary KIM-1 and cresecents, eighteen patients who were diagnosed as crescentic IgAN (defined as crescents in more than 50% of glomeruli on biopsy specimens) were also enrolled. The research was in compliance of the declaration of Helsinki and approved by the ethic committee of Tianjin Medical University General Hospital. Informed consent was obtained from each participant. For participants under the age of 18, written informed consents were obtained from the parents.

### Clinical and laboratory information

Clinical and laboratory data collected included the following: gender, age (years), time from onset (months), with/without prior infection, with/without gross hematuria, systolic and diastolic arterial pressure (mmHg), proteinuria (g/24 h), serum creatinine (μmol/L), estimated glomerular filtration rate (eGFR, ml/min/1.73 m^2^), serum IgA level (mg/dL) and serum Complement 3 level (mg/dL). eGFR was calculated with an equation developed by adaptation of the Modification of Diet in Renal Disease (MDRD) equation on the basis of data from Chinese chronic kidney disease patients [20].

### Evaluation of pathologic involvement

The minimum number of glomeruli in renal biopsy to allow the inclusion in the study was ten. The four pathologic variables of the Oxford classification were scored as follows [21,22]: Mesangial score: ≦0.5 (M0) or > 0.5 (M1); Segmental glomerulosclerosis: absent (S0) or present (S1); Endocapillary hypercellularity: absent (E0) or present (E1); Tubular atrophy atrophy/interstitial fibrosis: ≦25% (T0), 26–50% (T1) or >50% (T2). In addition, according to the recommendation of Oxford classification, there was a summary of the total number of glomeruli and the number with crescents and global glomerulosclerosis. Vascular lesions included hyaline change and wall thickening. Wall thickening was evaluated as the ratio of luminal diameter to outer diameter, and was considered to be present when the ratio was less than 0.5. Two pathologists who were blinded to patients’ data examined the slides separately. Differences in scoring were resolved by re-reviewing the biopsies and coming to consensus.

### Detection of urinary KIM-1

Urine samples were collected and centrifuged at 1500 g for 10 min to remove cellular components, and the supernatant was frozen at -80°C until use. Urine samples from 20 normal individuals were collected as controls. Urinary KIM-1 was measured by ELISA using a commercial kit (R&D Systems, DKM100) in accordance with the manufacturer’s guidelines. The lowest limit of detection for this assay was 0.046 ng/mL. The inter-assay coefficient of variation (CV) was 6.7%, and the intra-assay CV was 4.2%. The urinary KIM-1 level was standardized with urine creatinine (ng/mg). Adjusted urinary KIM-1 was expressed as urinary KIM-1 concentration/creatinine concentration (ng/mg).

### Statistical analysis

The quantitative data were expressed as mean ± standard deviation or median with interquartile range as appropriate. Differences of quantitative parameters with normal distribution between groups were assessed using the independent t-test. Differences of quantitative parameters with abnormal distribution between groups were assessed using the non-parametric test. Paired samples were assessed using the paired t-test. Differences of qualitative data were compared using chi-square test. The Spearman coefficient correlation was used to analyze correlation among various parameters. A P-value of less than 0.05 was considered to be statistically significant. Analysis was performed with SPSS statistical software package (version 17.0, Chicago, Ill, USA).

## Results

### Clinical and pathological characteristics of patients with mild IgAN

The clinical parameters of patients with mild IgAN were shown in Table 1.

**Table 1:** Clinical parameters of 51 patients with mild IgAN

**Characteristic**	
Male/female (n)	19/32
Age (years)	29.08 ± 7.96
Time from onset (months)	4(0.33, 84)
With/without prior infection	23/28
With/without gross hematuria	25/26
Systolic blood pressure (mmHg)	117.15 ± 12.05
Diastolic blood pressure (mmHg)	73.11 ± 9.05
Proteinuria (g/24 h)	0.56 ± 0.22
Serum creatinine (μmol/L)	74.75 ± 12.98
eGFR(ml/min/1.73 m^2^)	101.21 ± 24.50
Serum IgA (mg/dL)	335.47 ± 118.43
Serum Complement 3 (mg/dL)	94.42 ± 21.18

Data are expressed as mean ± SD or median with interquartile range.

The distribution of pathologic variables defined by the Oxford classification was as follows: M0/M1: 42/9 (82.4/17.6%); S0/S1: 45/6 (88.2/11.8%); E1/E2: 44/7 (86.3/13.7%) and T0/T1/T2: 46/5/0 (90.2/9.8/0.0%). The median percentage of glomeruli with crescents was 0.0% with an interquartile range between 0.0% and 35.0%. The median percentage of glomeruli with global glomerulosclerosis was 0.0% with an interquartile range between 0.0% and 40.0%. Among all 51 patients, 14 were with vascular lesions (hyaline change and/or wall thickening).

### Urinary KIM-1 in patients and normal controls

**Figure 1:**
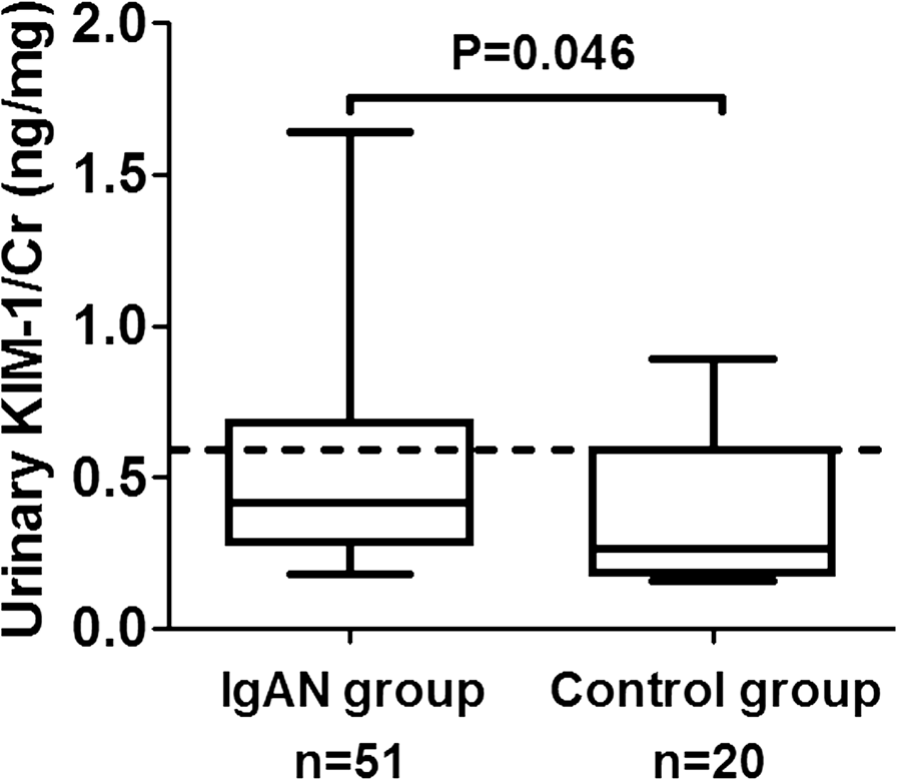
**Urinary KIM-1 in patients with mild IgAN and controls.** The dotted line represented the value of 95 percentile of the urinary KIM-1 of normal controls.

The urinary KIM-1 was significantly higher in patients with mild IgAN than that in normal controls [0.42 (0.18-1.64) vs. 0.27 (0.16-0.89) ng/mg, P = 0.046] (Figure 1). Since the level of urinary KIM-1 in normal controls was not normally distributed, the value of 95 percentile (0.52 ng/mg) was considered as a cut-off point. Patients were therefore divided into two groups according to the level of urinary KIM-1 [i.e. patients with elevated urinary KIM-1 (>0.52 ng/mg, n = 18) and patients without elevated urinary KIM-1(≤0.52 ng/mg, n = 33)].

### Comparison of EMST scores between patients with and without elevated urinary KIM-1

As shown in Table 2, there was no significant difference of all clinical parameters between patients with and without elevated urinary KIM-1. As for comparison of EMST scores, there was no significant difference of the distributions of the scores of mesangial hypercellularity (M), segmental glomerulosclerosis (S), and endocapillary hypercellularity (E) between two groups, while proportion of patients with T1 score of tubular atrophy/interstitial fibrosis (T) was significantly higher in patients with elevated urinary KIM-1 than that in patients without elevated urinary KIM-1.

**Table 2:** Comparison of the clinical and pathologic parameters between patients with and without elevated urinary KIM-1/Cr

**Variable**	**Without elevated urinary KIM-1/Cr (n = 33)**	**With elevated urinary KIM-1/Cr (n = 18)**	**P value**
Male/female (n)	11/22	8/10	0.547
Age (years)	30.03 ± 7.96	27.33 ± 7.89	0.252
Time from onset (months)	5(0.5, 60)	3(0.33, 84)	0.501
With/without prior infection	13/20	10/8	0.378
With/without gross hematuria	16/17	9/9	1.000
Systolic blood pressure (mmHg)	118.18 ± 10.95	115.28 ± 13.98	0.416
Diastolic blood pressure (mmHg)	74.24 ± 8.11	71.11 ± 10.51	0.241
Proteinuria (g/24 h)	0.52 ± 0.20	0.62 ± 0.24	0.149
Serum creatinine (μmol/L)	74.24 ± 13.09	75.69 ± 13.10	0.707
eGFR(ml/min/1.73 m^2^)	105.24 ± 23.19	104.56 ± 16.27	0.912
Serum IgA (mg/dL)	318.05 ± 111.82	374.27 ± 127.36	0.180
Serum Complement 3 (mg/dL)	93.73 ± 23.15	96.38 ± 19.04	0.774
Mesangial hypercellularity (M0/M1)	29/4	13/5	0.249
Segmental glomerulosclerosis (S0/S1)	28/5	17/1	0.405
Endocapillary hypercellularity (E0/E1)	29/4	15/3	0.686
Tubular atrophy/interstitial fibrosis (T0/T1/T2)	33/0/0	13/5/0	0.004*
With/without extracapillary proliferation (cresecents)	6/27	9/9	0.026*
With/without global glomerulosclerosis	13/20	10/8	0.378
With/without vascular lesions	6/27	8/10	0.057

*Being statistically significant. Since there was no patient with T2 in two groups, distribution of the variable of tubular atrophy/interstitial fibrosis was compared by chi-square test.

Since EMST scores were designed based on IgAN patients with proteinuria >0.5 g/24 h, 31 patients with proteinuria 0.5 ~ 1.0 g/24 h were then analyzed. For these patients, similar results were found. Proportion of T1 score was significantly higher in patients with elevated urinary KIM-1 than that in patients without elevated urinary KIM-1, and no difference of the scores of E, M and S was found between patients with and without elevated urinary KIM-1 (Table 3).

**Table 3:** Comparison of the EMST scores in patients with proteinuria 0.5 ~ 1.0 g/24 h

**Variable**	**Without elevated urinary KIM-1/Cr (n = 19)**	**With elevated urinary KIM-1/Cr (n = 12)**	**P value**
Mesangial hypercellularity (M0/M1)	17/2	8/4	0.174
Segmental glomerulosclerosis (S0/S1)	15/4	11/1	0.624
Endocapillary hypercellularity (E0/E1)	17/2	9/3	0.350
Tubular atrophy/interstitial fibrosis (T0/T1/T2)	19/0/0	9/3/0	0.049*

*Being statistically significant. Since there was no patient with T2 in two groups, distribution of the variable of tubular atrophy/interstitial fibrosis was compared by chi-square test.

### Comparison of glomeruli with cresecents and global glomerulosclerosis between patients with and without elevated urinary KIM-1

Besides EMST scores, proportions of glomeruli with extracapillary proliferation (cresecents) and global glomerulosclerosis between two groups were also compared. Proportion of glomeruli with cresecents was significantly higher in patients with elevated urinary KIM-1 than that in patients without elevated urinary KIM-1. There was no significant difference of the proportion of glomeruli with global glomerulosclerosis between two groups (Table 2).

### Comparison of vascular lesions between patients with and without elevated urinary KIM-1

Among 14 patients with vascular lesions, 8 patients were with elevated urinary KIM-1 and 6 patients were without elevated urinary KIM-1. Although the proportion of patients with vascular lesions was higher in patients with elevated urinary KIM-1 (44.4%) than that in patients without elevated urinary KIM-1 (18.1%), the difference was not significant (p = 0.057) (Table 2).

### Analysis of the relationship between urinary KIM-1 and glomeruli with cresecents

**Figure 2:**
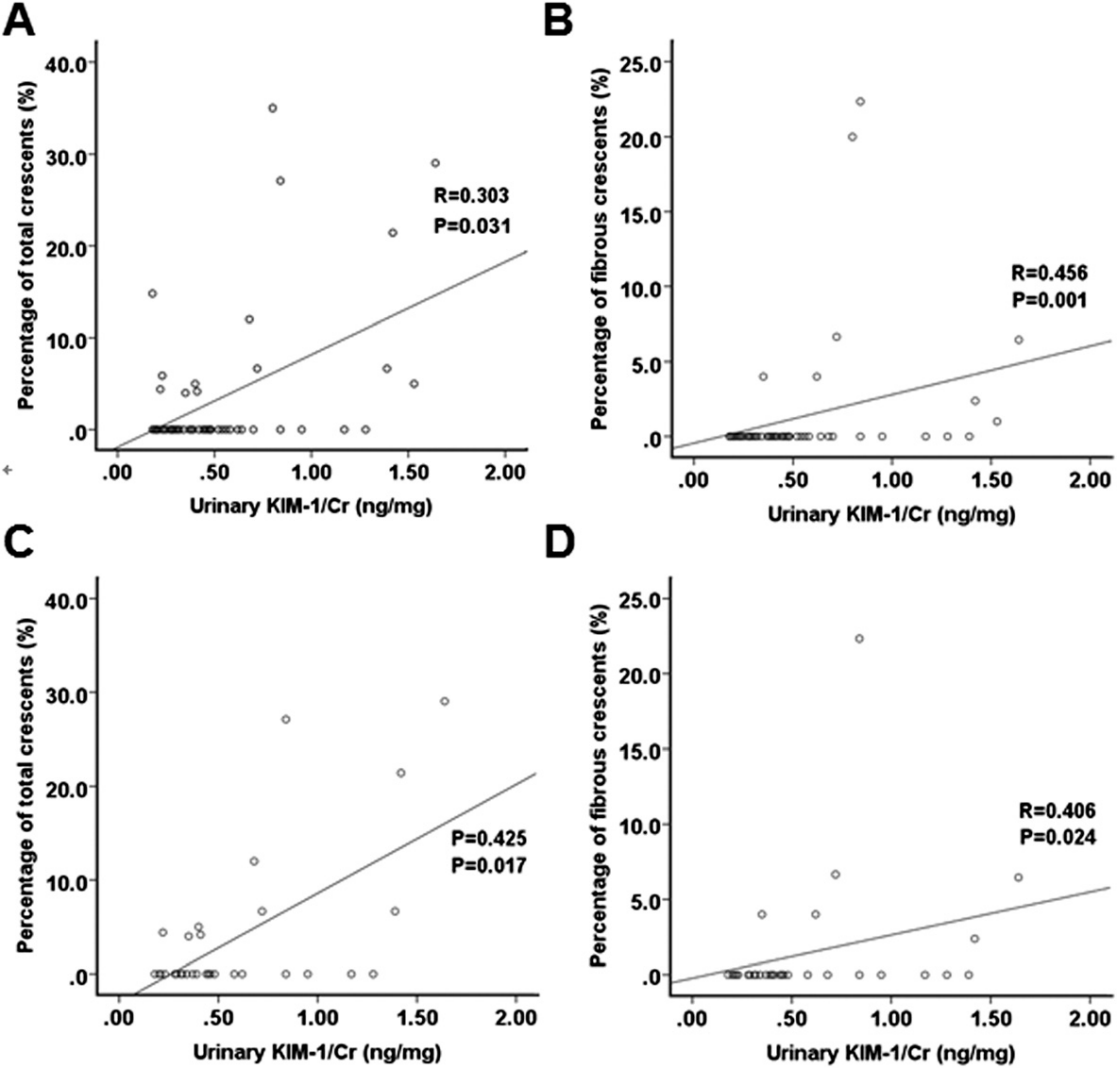
**Association between urinary KIM-1 and glomeruli with cresecents for patients with mild IgAN. A**: Association between urinary KIM-1 and the proportion of total cresecents for all patients with mild IgAN. **B**: Association between urinary KIM-1 and the proportion of fibrous cresecents for all patients with mild IgAN. **C**: Association between urinary KIM-1 and the proportion of total cresecents for the subgroup of patients with proteinuria 0.5 ~ 1.0 g/24 h. **D**: Association between urinary KIM-1 and the proportion of fibrous cresecents for the subgroup of patients with proteinuria 0.5 ~ 1.0 g/24 h.

Since there were more glomeruli with cresecents in patients with elevated urinary KIM-1, relationship between urinary KIM-1 and cresecents was further analyzed. The urinary KIM-1 of patients with cresecents was higher than that of patients without cresecents [0.68 (0.18-1.64) vs. 0.385 (0.18-1.28) ng/mg, P = 0.046]. The proportion of patients with T1 score was significantly higher in patients with cresecents (T0/T1/T2: 11/4/0) than that in patients without cresecents (T0/T1/T2: 35/1/0) (p = 0.022). Correlation analysis showed that for all 51 patients with proteinuria 0 ~ 1.0 g/24 h, urinary KIM-1 correlated with the percentage of glomeruli containing total crescents (Figure 2A). Further analysis indicated urinary KIM-1 correlated with the percentage of glomeruli containing fibrous crescents (Figure 2B), but did not correlate with the percentage of glomeruli containing cellular crescents or cellular/fibrous crescents. Similar results were found in patients with proteinuria 0.5 ~ 1.0 g/24 h (Figure 2C and D).

To further investigate whether the correlation found in patients with mild IgAN was valid in various stage of IgAN, correlation analysis was also made in 18 patients diagnosed as crescentic IgAN. For these patients, the percentage of glomeruli with crescents ranged from 50% to 95%. Unlike patients with mild IgAN, 72% patients with crescentic IgAN were with T2 score (T0/T1/T2: 2/3/13). Correspondingly, the urinary KIM-1 of patients with crescentic IgAN was significantly higher than that of patients with mild IgAN [1.34 (0.35-3.11) vs. 0.42 (0.68-1.64) ng/mg, P < 0.001]. However, no correlation between the percentage of glomeruli with crescents and urinary KIM-1 was found in patients with crescentic IgAN,

### Comparison of the response to treatment between patients with and without elevated urinary KIM-1

Twenty-two patients were followed up for 3 ~ 24 months. During follow up, 16 patients were treated with ACEis and/or ARBs and 7 patients were treated with immunosuppressive therapy (13.5%). The mean follow-up duration was 11.41 ± 6.06 months. After treatment, the follow-up proteinuria decreased significantly (0.53 ± 0.24 ng/mg) compared with the baseline level (ng/mg, 0.53 ± 0.24 vs. 0.62 ± 0.23 P = 0.039). There was no significant difference of the baseline proteinuria between patients with and without elevated urinary KIM-1 (g/24 h, 0.57 ± 0.23 vs. 0.70 ± 0.22, P = 0.218). There was also no significant difference of the follow-up proteinuria between patients with and without elevated urinary KIM-1 (g/24 h, 0.46 ± 0.19 vs. 0.64 ± 0.28, P = 0.09).

## Discussion

Urinary KIM-1 has been considered to be a biomarker for tubulointerstitial damage and repair and is not detected in severely damajged tubules [23]. Inconsistent results about the relationships between urinary KIM-1 and clinical parameters in IgAN have been derived by previous studies. The earliest study on the correlation between urinary KIM-1 and IgAN was made by Van Timmeren et al. [23]. The authors studied 102 patients with various kinds of kidney diseases including IgAN and found that urinary KIM-1 correlated negatively with renal function but not with proteinuria. However, there were only 10 patients with IgAN in that study. Subsequent studies focusing on patients with various stages of IgAN got different results [16,18]. In the current study, we found no correlation between urinary KIM-1 and proteinuria, as well as between urinary KIM-1 and renal function for IgAN patients with normotension, normal renal function and mild proteinuria, and the proportion of patients with elevated urinary KIM-1 was lower than previous studies [16,18]. We speculated that the heterogeneity of the selected patients might contribute to the differences of the results.

Oxford classification is the first specially-designed pathological system for evaluating the prognosis of IgAN. Among all 4 variables, tubular atrophy/interstitial fibrosis has been indicated to have the most predictive value by different validation studies [10-12]. In the current study, the patients with elevated urinary KIM-1 had more severe tubulointerstitial injury (5/18 patients with elevated urinary KIM-1 had T1 score, while no patient with normal urinary KIM-1 had T1 score). Although no difference of the response to treatment between patients with and without elevated urinary KIM-1 was observed during a short-term follow-up, it seems that the long-term prognosis of the patients with elevated urinary KIM-1 should be paid more attention.

Although KIM-1 is a sensitive and specific biomarker for the tubulointerstitial damage, elevated urinary KIM-1 can be observed not only in the kidney diseases characterized mainly by tubulointerstitial damage, but also in that characterized exclusively by globular damage (such as minimal change disease) [23]. In these diseases, tubulointerstitial damage should be due to reabsorption of protein. So urinary KIM-1 is a marker of the disease activity, however when tubules and interstitium are extremely damaged, KIM-1 will disappear. In the current study, significant correlation between urinary KIM-1 and crescent formation was found in patients with mild IgAN. Previous studies indicated more than 20% of IgAN patients could be found to have crescents on the initial biopsy [24-26]. Poor prognosis of crescentic IgAN has been generally recognized by clinicians [25]. Coexistence of the cellular, fibrocellular and fibrous crescents reflects the process of slow progression of IgAN. Although crescent formation was not demonstrated to be an independent risk factor of ESRD in the Oxford classification, the significance of crescent formation in IgAN should not be neglected, because patients with rapidly progressive kidney failure were excluded when developing the Oxford classification.

IgAN is a kind of glomerulopathy and the tubular/interstitial injury should emerge following the glomerular injury. The chronic glomerular damage causes local microcirculation barrier, and then causes kidney ischemia and damages the tubules and interstitium. Although no correlation between crescents and urinary KIM-1 was found in 18 patients with crescentic IgAN, the urinary KIM-1 level of these patients was higher than that of patients with mild IgAN and the tubular/interstitial injury was much more severe (72% patients with crescentic IgAN were of T2 score). Thus, we speculated that crescents formation might be able to cause tubular/interstitial injury and then induce the expression of KIM-1 indirectly. It was noteworthy that for patients with mild IgAN, it was the fibrous crescent not the cellular crescent that correlated with urinary KIM-1. Fibrillation of crescents means the acute damage of glomeruli turn to be chronic and irreversible, and the microcirculation of interstitium will be injured inevitably. Thus, the correlation between fibrous crescents and urinary KIM-1 found in patients with mild IgAN might be a reflection of the ischemic injury of tubules and interstitium caused by the irreversible glomerular damage.

Although the exact reason is still unclear, previous studies indicated that vascular lesions in IgAN were very common [27,28]. Since vascular lesions can influence the blood supply to the glomeruli, tubules and interstitium and cause more damage to the glomerular and tubulointerstitial tissues, it might also influence the expression of KIM-1. In the current study, no significant difference of the incidence of vascular lesions between patients with and without elevated urinary KIM-1 was found. However, since the p-value of the comparison had reached 0.057, we thought that the result might be reversed when the number of patients could be enlarged enough.

### Limitations

There are some limitations to the present study. First, According to Oxford classification, the histologic lesions of biopsy of patients with clinically mild IgAN were relatively small. The proportion of patients with T1 was low and no patient was found to be with T2. This might have an influence on the analysis of the relationship between urinary KIM-1 and the tubulointerstitial injury. Second, urinary KIM-1 was measured at a single time-point and it is unclear whether the level of urinary KIM-1 fluctuates on different occasions. Finally, since the time of follow-up is short, we could not judge the long-term outcome of these patients at present. A large-scale analysis with a long time follow-up is warranted.

## Conclusions

In conclusion, urinary KIM-1 is a reflection of tubulointerstitial injury. For patients with IgAN without clinical risk factors, high urinary KIM-1 level is related to relatively severe pathologic involvement on renal biopsy. There was no difference of the response to treatment during a short time follow-up between patients with and without elevated urinary KIM-1. However, since IgAN tends to progress slowly, a long-term follow-up is still needed.

## Competing interests

The authors have no competing interests.

## Authors’ contributions

XPC participated in coordination, wrote the draft manuscript. WL made the pathological confirmations of IgAN, reviewed medical records, and participated in data analysis. SWY carried measurement of urinary KIM-1. TSL participated in design, helped to collect sample and analyzed statistic data. GDM participated in study design, enrolled patients, and managed samples. YTK participated in design and manage the research project. LS participated in design and coordination, and helped to draft the manuscript. All authors read and approved the final manuscript.

## Pre-publication history

The pre-publication history for this paper can be accessed here:

http://www.biomedcentral.com/1471-2369/15/107/prepub
